# The role of the lymphotoxin-β receptor (LTβR) in hepatocyte-mediated liver regeneration

**DOI:** 10.1186/2047-783X-19-S1-S3

**Published:** 2014-06-19

**Authors:** Kristina Behnke, Ursula R Sorg, Diran Herebian, Dieter Häussinger, Verena Keitel, Klaus Pfeffer

**Affiliations:** 1 Institute of Medical Microbiology and Hospital Hygiene, Heinrich Heine University, 40225 Düsseldorf, Germany; 2 Department of General Pediatrics, Neonatology and Pediatric Cardiology, Heinrich Heine University, 40225 Düsseldorf, Germany; 3 Clinic of Gastroenterology, Hepatology and Infectious Diseases, Heinrich Heine University, 40225 Düsseldorf, Germany

## Background

The lymphotoxin beta receptor (LTβR) is a prototypic member of the TNF/TNFR superfamily and has diverse functions in regulating immune responses against pathogens, in organogenesis and maintenance of structural integrity of secondary lymphoid tissues [1]. Mice deficient in LTβR (LTβR^-/-^) show impaired resistance to intracellular pathogens and defects in peripheral lymphoid tissues [2,3]. Importantly, LTβR^-/-^ mice also exhibit markedly reduced survival after partial (70%) hepatectomy (PHx) [4], compared to WT controls. Liver mass is tightly regulated to 5% of the body weight and the liver retains a remarkable capacity for regeneration in response to acute injury. Loss of at least 30% of liver mass leads to synchronized proliferation of normally quiescent mature hepatocytes (compensatory hyperplasia) until physiologic liver mass is regained. Recently, it has been shown that lymphotoxins required for liver regeneration are produced by T cells immigrating into the liver during the regeneration process [5]. During this regeneration process, it is mandatory that the liver continues to perform its essential functions, such as protein synthesis, glycogen storage and bile secretion. There is evidence, that nuclear receptor-dependent bile acid signaling is required to initiate efficient liver regeneration [6] and that signaling through the TNFRp55, another core member of the TNF/TNFR superfamily is an essential part of early liver regeneration [7]. Interestingly, signaling pathways through TNFRp55 and LTβR share downstream signaling components. However, the molecular mechanisms orchestrated by LTβR and TNFRp55 signaling remain incompletely defined.

## Results

Our data confirms that following PHx LTβR^-/-^ mice have a decreased survival rate compared to WT mice (62 % vs. 90 %, respectively). Surviving LTβR^-/-^ mice show a rate of liver regeneration comparable to WT animals. Post PHx H&E stained liver sections of LTβR^-/-^ animals showed higher numbers and larger areas of necrosis in the liver tissue, increased vacuolisation of hepatocytes, oedematous changes in the portal fields and dilated lymhatic vessels. ELISAs and clinical chemistry analysis show an altered cytokine expression profile in LTβR^-/-^ animals and several serum proteins appear to be deregulated, e.g. a significant increase in alkaline phosphatase in LTβR^-/-^ animals 24 and 48 h post PHx could be observed. On the other hand, ALT and AST levels do not differ between LTβR^-/-^ and WT animals. Total bile acid levels as well as the bile acid profile, as determined by UPLC-MS/MS, change markedly in both cohorts after PHx and differ between WT and LTβR^-/-^ animals, with the latter showing indications of cholestasis. This difference in bile acid metabolism is supported by quantitative RT/PCR data which shows variations in expression levels of various transporters and regulatory proteins involved in bile acid homeostasis between the two cohorts. Interestingly, expression of the LTβR gene on *in vitro* outgrown cholangiocytes of untreated WT animals could be demonstrated. Microarray analysis of WT and LTβR^-/-^ mice identified a panel of differentially expressed genes; most prominent among these was a markedly decreased expression of murinoglobulin-2, a proteinase inhibitor of the α2-macroglobulin family, in LTβR^-/-^ mice.

## Conclusions

Our findings demonstrate that a deficiency in LTβR signalling leads to substantial changes in liver morphology, gene expression, cytokine levels and other serum parameters after PHx. The various bile acids analyzed are differentially regulated after PHx in WT animals and this regulation is disturbed in LTβR^-/-^ animals after PHx. This markedly different bile acid profile in LTβR animals after PHx that seems to be associated with cholestasis indicates that bile acid homeostasis may play a key role in determining survival after 70 % PHx in rodents. It is not yet known how LTβR signaling pertains to bile acid metabolism, and the detailed mechanisms of this interaction are in the focus of further studies. In conclusion, these results emphasize the importance of LTβR signaling for efficient liver regeneration and may provide further insights into this complex process.
